# Interesting Facts

**Published:** 1863-04

**Authors:** 


### INTERESTING FACTS.

Raphael and Luther were both born in the year 1483. The former died in 1520, the same year with Da Vinci.—Spencer was born in 1553, the year in which Latimer died.—Sir Walter Raleigh and Hooker were also born within a few months of Spencer.—Shakspeare and Galileo were both born in 1564, the year in which Luther and Calvin and Roger Ascham died.—Galileo was born the day Michael Angelo died, and died the day Newton was born.—Newton made one of his first experiments at the age of sixteen, on September 3d, 1658, the day of the great storm, when Cromwell died.—Cromwell was born in 1599, the year in which Spencer died.—Izaak Walton, Newton, and Tasso, all died in 1593.—Claude Lorraine and Poussin, the artists, were born in 1600, the year in which Hooker died.—Claude and Murillo died in the year 1682.—Milton, Clarendon, and Fuller, were all born in 1608. The two former died in the same year, 1674, and the year in which Watts was born.—Shakspeare and Pocahontas died in the same year, 1616.—Raleigh died in 1618, the year in which the famous Synod of Dort was formed.—Bunyan was born in 1628, the year in which Decker died, and died in 1688, the year Pope was born.—Dryden was born in 1631, the year in which Donne died, and died in 1700, the year in which Thomson and Blair were born.—Galileo, Guido, and Boyle, all died in 1642.—Burnet, the historian, was born in 1643, the year in which Hampden died.—Rollin and Fuller died the year Defoe was born, 1661.—Swift was born in 1667, the year Jeremy Taylor died.—Locke and Sir Christopher Wren were both born in 1632.—Bolingbroke and Addison were both born in 1672, two years before Milton died. —Defoe died in 1713, the year Sterne was born.—Burnet died in 1714, the year Whitefield and Shenstone were born.—Leibnitz died in 1716, the year Garrick and Gray were born.—Penn died in 1718, the year Putnam and Brainard were born.—Sir C. Wren died in 1723, the year in which Blackstone and Reynolds were born.—Cowper was born in 1731.—Goldsmith was born in 1729, the year in which Steel died.—Gibbon, Smollett, Collins, and Akenside, were all born in 1721.—Gibbon and Akenside both died in 1794, the same year Witherspoon died.—Watts and Thomson died in 1748.—Voltaire and Pitt in 1778.—Christopher Wren, in 1773, the year Priestley and Coleridge were born. George Washington, Patrick Henry, and Howe, all died in 1799.—Cromwell and Hampdon, who were cousins, both took passage in a vessel that lay in the Thames bound for North America, in 1637. They were actually *on board* when an order of council appeared by which the ship was prohibited from sailing.—Goethe was at one time, also, on the brink of crossing the ocean for America.—So was Robert burns.—A scheme of Pautisocracy in 1795, came near bringing Southey, Coleridge, Lovell, and Burnet to America.—Chaucer was the first of that long array of poets buried in Westminster Abbey, in 1400.—The body of Dryden was deposited in the grave of Chaucer, just three centuries after his burial, in the year 1700.—Goldsmith died two thousand pounds in debt.—As proof of the wonderful memory of Thomas Fuller, it is said that he could repeat five hundred unconnected words after twice hearing them, and recite the whole of the signs in the principal street of London, after once passing through it and back again.—Locke was banished as a traitor, and wrote his "Essay on the Human Understanding," sheltering himself in a Dutch garret.—Homer sang his own ballads.—Virgil was so fond of salt that he seldom went without a boxful in his pocket.—Addison, who is acknowledged to have been one of the most elegant writers that ever lived, was awkwardly stupid in conversation.—Handel was such a miser, that he was frequently known to wear a shirt a month to save the expense of washing.—It is said that Dryden was always cupped and physicked previous to a grand effort at tragedy.—He was a firm believer in astrology.—It is said that Pitt required a great deal of sleep, seldom being able to do with less than ten or eleven hours.—Butler did not become an author until he was fifty years old.—Richardson, author of "Pamela," etc., did not begin to write till he was almost fifty years of age.—Robert Ferguson died in an insane asylum.—The wife of Beattie, the poet, became insane and was confined in an asylum for some years.—The first wife of Southey died insane.—Chatterton put a period to his own life at the age of eighteen.—Coleridge was for many years addicted to the use of opium.—Sir William Jones was the master of twenty-eight languages.—The father of Henry Kirke White was a butcher, as was also that of Cardinal Wolsey and the poet Akenside—White was apprenticed to a stocking weaver.—Montgomery, at the age of fourteen, to a shopkeeper.—Crabbe was the son of a salt-master, or collector of salt duties.—Coleridge was the son of a vicar.—Samuel Rogers was a banker by profession.—The father of Charles Lamb was servant and friend to one of the batchelors of the Inner Temple.—Campbell was born in the sixty-seventh year of his father's age, and was the youngest of ten children.—Keats was born in a livery stable, and was apprenticed at fifteen to a surgeon.—Alexander Wilson, the distinguished naturalist, was brought up to the trade of a weaver, but afterwards preferred that of a pedlar and after that was a schoolmaster.—Robert Dodsley, who was the projector of the "Annual Register" in which Burke was engaged, and who was the first to collect and republish the "Old English Plays" which formed the foundation of the "National Drama," raised himself from the low condition of a livery servant, to be one of the most respectable and influential men of his time.—Canova was the son of an old quarryman, and originally a laborer.—Thorwaldsen, of a carver of ship heads.—Samuel Rogers was fixed in his determination to become a poet by the perusal of "Beattie's Minstrels," when only nine years of age.—The Rev. William Lisle Bowles enjoys the distinction of having delighted and inspired the genius of Coleridge.—The study of "Percy's Reliques of English Poetry" gave the first impulse to the genius of Sir Walter Scott. He has also stated that the rich, human, pathetic tenderness and admirable tact of Miss Edgeworth's "Irish Portraits," led him first to think that something could be done, or attempted, for his own country, of the same kind as she had so fortunately achieved for Ireland.—During the last six years of the life of Chalmers, his daily modicum of original composition was completed before breakfast, written in short hand, and all done in bed.—Milton frequently composed lying in bed in the mornings; but when he could not sleep, and lay awake whole nights, not one verse could he make. He would sometimes dictate forty lines in a breath, and then reduce them to half the number.

